# Microstructural Analysis and Radiological Characterization of Alkali-Activated Materials Based on Aluminosilicate Waste and Metakaolin

**DOI:** 10.3390/gels11010057

**Published:** 2025-01-10

**Authors:** Nataša Mladenović Nikolić, Aleksandar Kandić, Jelena Potočnik, Nemanja Latas, Marija Ivanović, Snežana Nenadović, Ljiljana Kljajević

**Affiliations:** 1 Department of Materials, “Vinča” Institute of Nuclear Sciences—National Institute of the Republic of Serbia, University of Belgrade, 11000 Belgrade, Serbia; natasa.nikolic@vin.bg.ac.rs (N.M.N.); marija@vin.bg.ac.rs (M.I.); msneza@vin.bg.ac.rs (S.N.); 2 Department of Nuclear and Plasma Physics, “Vinča” Institute of Nuclear Sciences—National Institute of the Republic of Serbia, University of Belgrade, 11000 Belgrade, Serbia; akandic@vin.bg.ac.rs; 3 Department of Atomic Physics, “Vinča” Institute of Nuclear Sciences—National Institute of the Republic of Serbia, University of Belgrade, 11000 Belgrade, Serbia; jpotocnik@vin.bg.ac.rs (J.P.); nemanja.latas@vin.bg.ac.rs (N.L.)

**Keywords:** fly ash, wood ash, metakaolin, gel structure, natural radioactivity

## Abstract

The formation of an aluminosilicate gel structure made of alkali-activated materials (AAMs) was conducted through an alkali-activation reaction of the solid precursors (fly ash, metakaolin, and wood ash). Fly and wood ash are by-products of the burning process of coal and wood, respectively. Alkali-activated materials of aluminosilicate origin, made from the different ashes, fly and wood, are very attractive research targets and can be applied in various technological fields due to their thermal stability, resistance to thermal shock, high porosity, high sustainability, and finally, low energy loss during production. In this paper, we evaluate physico-chemical properties, microstructure, and radiological environmental impacts when wastes that contain elevated levels of naturally occurring radionuclides (NORs) such as fly ash and wood ash are made into “green cements” such as AAMs. The determination of radionuclide content was performed by means of gamma-ray spectrometry. Results showed that the AAMs have a lower value in the activity concentration of radionuclides than raw materials. The external absorbed gamma dose rate was 74.7–107.3 nGy/h, and the external radiation hazard index values were in range of 0.445–0.628 Bq/kg. The results of the activity concentration measurements for alkali-activated materials indicate the potential of their safe application in building construction. In terms of the structural characterizations, the obtained alkali-activated materials were examined using XRD, DRIFT, FESEM, and TEM analyses.

## 1. Introduction

The rapid growth of industrial development has led to a significant increase in waste production, making environmental management a critical challenge. The improper storage of fly ash, an industrial by-product, has contributed to land contamination issues. However, these by-products hold potential as secondary raw materials in construction, supporting the circular economy and helping to prevent environmental degradation. One promising solution is the development of alkali-activated materials (AAMs), which can serve as alternatives to traditional cements. AAMs are aluminosilicate-based materials that offer numerous advantages, including reduced environmental pollution and lower CO_2_ emissions during production [1,2]. These materials are highly valued for their excellent mechanical strength, durability, thermal stability, and fire resistance. Furthermore, their manufacturing process requires less energy, making them a sustainable choice for modern construction needs [3,4].

Alkali-activated materials (AAMs) are synthesized using solid waste materials as precursors, such as fly ash (FA) and wood ash (WA), which are by-products of coal and wood combustion, respectively. The improper disposal of these waste materials, particularly fly ash, can lead to significant environmental challenges [5]. The formation mechanism consists of three to four stages such as the dissolution of raw materials, the polymerization of silica and alumina (gel phases I and II), condensation through the process of gelation, and reorganization. The time required for the supersaturated aluminosilicate solution to form a continuous gel varies significantly depending on the type of raw material, the composition of the alkali-activated solution, and the process conditions. Geopolymers made from metakaolin are consistent and predictable in both their preparation and property development. Although particle dispersion during mixing slightly affects the rheology and reactivity of the gel structure, the reaction of metakaolin-based geopolymers is not significantly influenced by variations in the raw material’s surface area. However, the extent of the reaction is impacted by the soluble silicate and alkali content in the activating solution [6]. On the other hand, fly ash is an industrial by-product without a clearly defined starting composition. It primarily consists of silicon, aluminum, and iron oxides, with Class C ashes [7] containing notable amounts of calcium. Fly ash particles are typically spherical but heterogeneous, containing both glassy and crystalline phases, such as mullite and quartz. Due to this variability, attention is required to optimize mixture designs when using fly ash for geopolymer production.

Candamano et al. (2017) investigated the partial replacement of metakaolin with wood ash and found that it improves the material’s workability. This improvement is attributed to the enhanced dissolution of metakaolin in mixtures with higher alkalinity due to the presence of wood ash. However, materials containing wood ash exhibit a more porous and uneven matrix, leading to higher sorptivity. The altered pore structure, combined with increased drying shrinkage, suggests that wood ash influences the material’s physical properties [8]. These findings indicate that wood ash can serve as a viable partial substitute in alkali-activated metakaolin-based materials. According to our previous research [9], wood ash contains a high percentage of CaO, and the incorporation of calcium in various forms during the synthesis of AAMs affects the reaction pathway. This results in increased material strength, expanding the potential applications of AAMs in the construction sector [10]. Duxon et al. (2007) emphasized the importance of calcium content in raw materials in defining the reaction pathway and the physical characteristics of the resulting geopolymer [11]. In particular, the alkalinity affects the stability of several calcium-containing residues [12]. Lee and van Deventer (2007) [13] observed that minor insertions of soluble calcium to a fly ash-based geopolymer importantly diminished the setting time of geopolymers. Construction materials as well as these other materials contain natural radioisotopes [14]. The dose of exposure to radiation largely depends on the physical and chemical properties of radionuclides. The effects of radiation depend on various factors, such as the variety of radiation, the energy extent, and whether it influences the human body externally or internally [15].

The main natural radionuclides of the Earth are ^238^U, ^232^Th and its progeny, and ^40^K, which constitute the primary sources of external gamma radiation exposure to humans. The accumulation of radioactive pollutants exerts dangerous effects on the population, animals, and environments [16]. Waste produced from burning coal or other similar materials in thermal power plants contains trace elements as well as specific concentrations of radionuclides. It is necessary to learn the radiological characteristics of combustion by-products to estimate their capacity for advanced uses, such as in construction. Insight into their radiological characteristics enables greater verification of radiation exposure levels [17,18].

Coal contains significant amounts of natural radionuclides, and its combustion in power plants generates ash that poses environmental and health risks to surrounding populations [19]. Ignjatiović et al. [20] found that the natural radionuclide content in all tested concrete samples remained below the recommended threshold. Alkali-activated materials (AAMs) derived from aluminosilicate sources, such as various types of ash and metakaolin, exhibit desirable properties that make them suitable for numerous technological applications. These include thermal stability, resistance to thermal shock, high porosity, large specific surface area, sustainability, and low energy requirements during production. AAMs intended for the construction sector can be tailored by modifying synthesis parameters, such as the type of precursor or the molarity of the alkaline activator, facilitating a “green” and efficient production process.

It has been shown in our earlier studies [9] that the alkaline activation of aluminosilicate precursors affects the reduction in radionuclide activity more compared to the precursors. The aim of this research, in addition to examining the structural characteristics of AAMs, is to investigate the possibility of using fly and wood ash in the construction sector from a radiological point of view.

## 2. Results and Discussion

The process of geopolymerization involves the formation of a gel-like network structure through the chemical activation of aluminosilicate materials, typically in the presence of an alkaline solution. The primary mechanism begins with the dissolution of the raw materials, such as metakaolin, fly ash, in the alkaline medium, resulting in the release of silica (SiO_2_) and alumina (Al_2_O_3_) species into the solution. These species then undergo polymerization reactions, forming a gel network composed of Si-O-Al bonds. As the reaction progresses, the gel structure become more interconnected, creating a rigid, solid matrix that binds the aluminosilicate particles together. The formation of this geopolymer gel is strongly influenced by factors such as the pH of the solution, the Si/Al ratio, and the presence of additional ions or compounds like calcium, which can modify the gel structure and its properties. The final product is a stable, durable material that exhibits similar characteristics to those of traditional cement, but with a lower environmental impact. The investigation of geopolymerization was focused on the specific and unique phenomena that occur under reaction conditions, including low-temperature synthesis, highly concentrated slurries, the formation of an amorphous three-dimensional gel structure, and varying raw materials for creating amorphous products [11,21].

### 2.1. XRF Analysis

Table 1 presents the chemical composition of the AAMs, which was determined through X-ray fluorescence (XRF) analysis. The chemical composition of the solid precursors, FA, MK, and WA, was published in our previous article [22].

It can be observed that the AAMs containing WA have a two to three times higher CaO content compared to AFA_50_MK_50_. This is, of course, directly related to the CaO content in WA as a raw material. The results of these measurements can be found in our reference [9]. Regarding the SiO_2_/Al_2_O_3_ ratio, it ranges from 2.30 (AWA_10_MK_90_) to 2.75 (AWA_10_FA_90_), while the samples AWA_10_FA_45_MK_45_ and AFA_50_MK_50_ have approximately the same values for this ratio, which is expected, with values of 2.50 and 2.62, respectively.

### 2.2. DRIFT Analysis

**Figure 1:**
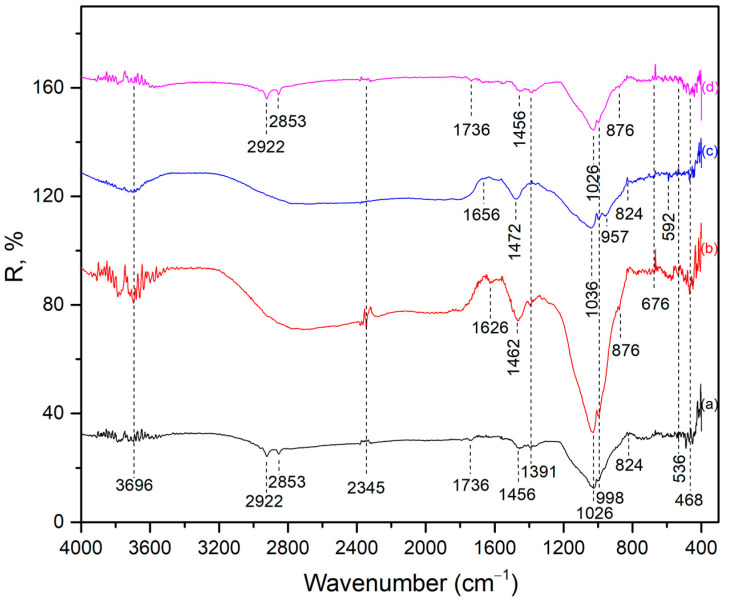
DRIFT spectrum of alkali-activated materials: (a) AWA_10_FA_90_, (b) AWA_10_MK_90_, (c) AFA_50_MK_50_, and (d) AWA_10_FA_45_MK_45_.

Figure 1 describes the DRIFT spectrum of the investigated AAMs.

The presence of wide bands in the range of the OH-group vibrations was noted in the DRIFT spectrum. The band around 3700 cm^−1^ was derived from the regularly distributed OH group in the structure, i.e., the H-O-H bending vibration. The small peak at wave number ~1652 cm^−1^ indicates adsorbed or associated water molecules or corresponds to the O-H stretching vibration [23]. Vibrations associated with symmetric and asymmetric stretches of C-H in the methyl and methylene groups, present in the spectrum, were found at wavelengths 2922 cm^−1^ and 2853 cm^−1^ [24,25], respectively. A small peak at 1736 cm^−1^ indicated CO_3_^2−^ vibrations, and it was noticeable in AWA_10_FA_90_ and AWA_10_FA_45_MK_45_ samples [26]. Its presence was not noted in the other two samples, but undoubtedly, this functional group is present in them as well and was incorporated or overlapped by some other vibration. The characteristic C=O stretching asymmetric vibrations and carbonate vibrations at 1456–1472 cm^−1^ were expected due to the possibility of carbon dioxide formation [23]. Furthermore, vibrational peaks situated in the 1026–1036 cm^−1^ region are related to the Si-O-T asymmetric stretching vibration (where T represents aluminum/silicon) functional groups, which describe the formation of an amorphous gel to semi-crystalline aluminosilicate materials, while a vibration at 468 cm^−1^ indicated Si-O bonds [27]. These vibrations were the main characteristic of the alkali-activated materials and were shifted at lower wavenumbers than the raw materials [27]. Additionally, a vibration at 957 cm^−1^ can be observed in AFA_50_MK_50_, which is characteristic of a Si-O-T stretching symmetric vibration (T = Si, Al) [28]. The refraction peak observed within the wavelength range of 532–876 cm^−1^ can be attributed to the Al-O stretching vibrations occurring within the [AlO_6_] octahedral, which is consistent with the presence of the mineral phase mullite in the fly ash composition, which does not dissolve under the applied conditions, but has the role of a strengthening and refractory phase.

### 2.3. XRD Analysis

**Figure 2:**
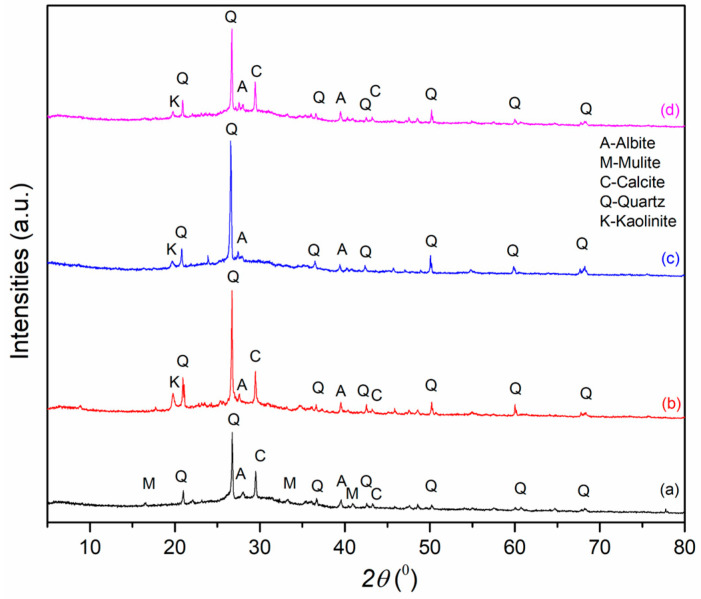
XRD diffractogram of alkali-activated materials: (a) AWA_10_FA_90_, (b) AWA_10_MK_90_, (c) AFA_50_MK_50_, and (d) AWA_10_FA_45_MK_45_.

XRD diffractograms of alkali-activated materials derived from metakaolin and fly and wood ashes are shown in Figure 2.

In very concentrated solutions, which frequently produce amorphous materials, ionic species are not fully hydrated. Rather than being quite surrounded by water molecules, some position in the hydration capsule of alkali cations like Na^+^ are taken possession of by silicate anions, resulting in ion pairing. This interaction, along with steric barrier during precipitate formation, inhibits long-range structural ordering and produces the material as an amorphous gel state. This is characteristic of geopolymeric gels, as presented in Figure 2. The reaction mechanisms that lead to the formation of the geopolymer gel phase also play a role in the appearance of crystalline phases. In addition, research indicates that even patterns classified as “X-ray amorphous” may express some degree of short-range structural arrangement, as confirmed by support from the literature [29]. In addition to the amorphous gel phase in the geopolymer gel, crystalline or semi-crystalline phases are observed, which originate from the precursors or result from partial crystallization [11,22].

X-ray diffraction results reveal that geopolymer samples AWA_10_FA_90_ and AWA_10_MK_90_ (Figure 2 (a) and 2 (b), respectively) activated with an alkali activator do not show significant differences in mineralogical composition. However, the first samples (a) exhibit slightly more pronounced mullite peaks (PDF No. 01-070-975), consistent with the literature findings [30], while in the second sample (b), a phase of kaolinite can be identified (PDF No. 01-072-5860), which originates from kaolin. Sample AWA_10_FA_90_ (Figure 2 (a)), an alkali-activated raw mixture of WA_10_FA_90_, exhibits the highest background intensity, between 20° and 40° 2θ, indicative of its amorphous-phase content. The baseline (diffractogram b) is shifted and its range is narrower than that of the previous sample (diffractogram a), suggesting a decreased presence of the amorphous phase, which was observed in the 15°-to-40° 2θ range. Additionally, peaks corresponding to calcite (PDF No. 01-078-4616) are identified in the AAMs which contain wood ash. The dominant crystalline phase detected in AFA_50_MK_50_ (Figure 2 (c)) and AWA_10_FA_45_MK_45_ (Figure 2 (d)) is quartz (PDF No. 00-033-1161), but the presence of calcite (PDF No. 01-078-4616) can be also observed, as an additional effect of WA in the AWA_10_FA_45_MK_45_ sample. Additionally, albite (PDF No. 01-078-1995) is present in all investigated samples.

### 2.4. FESEM/EDS

**Figure 3:**
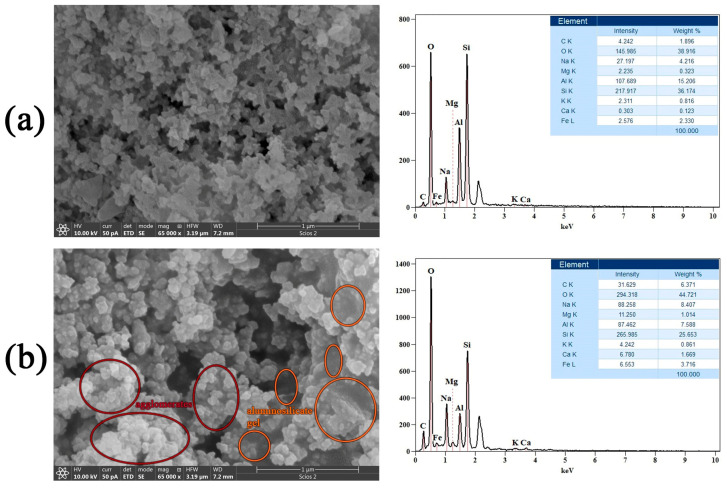
FESEM/EDS micrographs of AAMs, (**a**) AMK_100_ and (**b**) AFA_100_, together with the corresponding EDS spectra.

The morphology of the synthesized samples was examined using FESEM/EDS. The results are shown in Figure 3 (left side). As for the basic AMK_100_ and AFA_100_, there is no considerable difference in the morphology of the samples. The high porosity and somewhat different shapes of the particles in these two samples are characteristic of these two materials. EDS analysis was performed to evaluate the chemical composition of the samples, and the results are shown in Figure 3 (right side). EDS spectra were recorded within the energy range of 0.1–10 keV. The results indicate that the examined materials mainly consisted of oxygen (O), aluminum (Al), silicon (Si), carbon (C), potassium (K), and sodium (Na). Furthermore, some additional peaks were detected, including those related to magnesium (Mg), iron (Fe), and calcium (Ca).

The prepared AAM samples were analyzed to investigate possible changes in their morphology due to different mixtures of precursors, i.e., how the addition of WA affects FA and MK individually, as well as its effect on alkali-activated mixtures of these two precursors (FA and MK) in an equal ratio.

**Figure 4:**
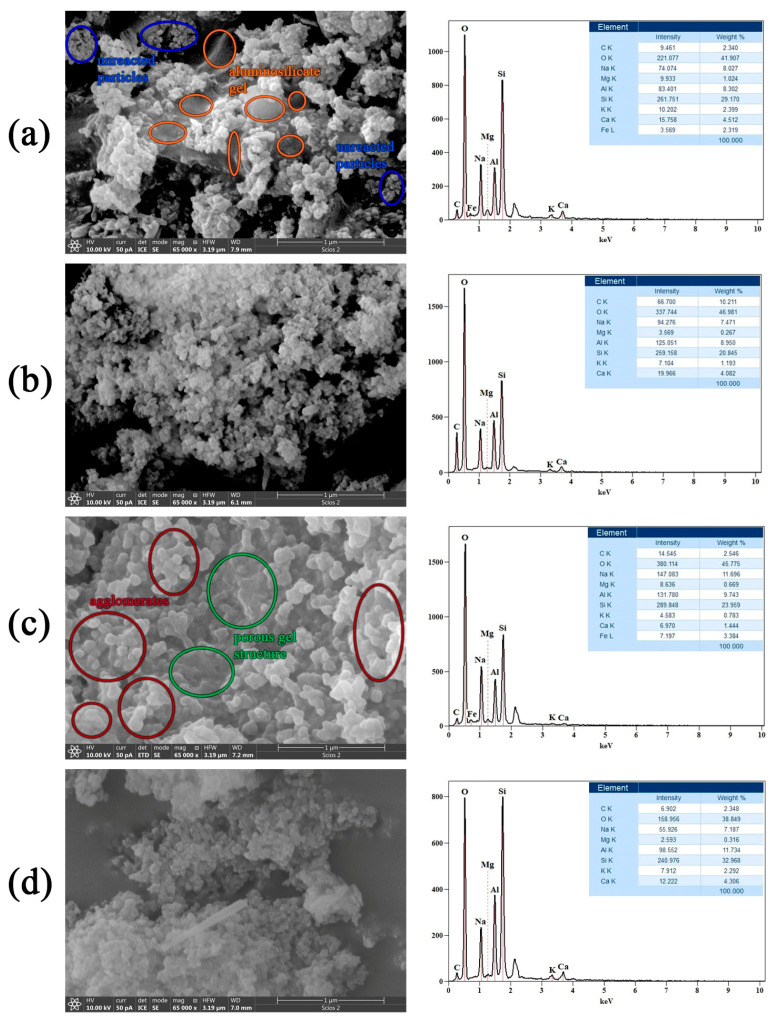
FESEM/EDS micrographs of AAMs, (**a**) AWA_10_FA_90_, (**b**) AWA_10_MK_90_, (**c**) AFA_50_MK_50_, and (**d**) AWA_10_FA_45_MK_45_, and the corresponding EDS spectra.

The SEM micrographs shown in Figure 4 (left side) display the microstructures of wood ash/metakaolin (Figure 4a), wood ash/fly ash (Figure 4b), metakaolin/fly ash (Figure 4c), and wood ash/metakaolin/fly ash (Figure 4d) alkali-activated materials. The microstructures presented in Figure 4a,b,d show a greater extent of heterogeneity introduced by the precipitation of high-Ca phases, originating from wood ash, throughout the geopolymer gel matrix. The formation of calcium compounds in geopolymers is greatly dependent on the pH and Si/Al ratio [12,31]. Fly ashes generally contain appreciable levels of iron in various forms, either as a network former or a network modifier, in the glassy phases [32]. This is a similar effect to that of calcium with regard to the precipitation of calcium hydroxide, which removes hydroxide ions from the solution phase, and affects the setting behavior and material properties. In Figure 4a, the gel structure is observed to have unevenly distributed particles of irregular shapes and sizes below 1 micron on its surface. The microstructure of the surface of the AWA_10_MK_90_ sample, shown in Figure 4b, is entirely different from the surface of the sample in Figure 4a. A large number of very fine particles, smaller than 100 nm, are grouped (agglomerated) and densely packed across the entire surface. Alkali activation of an equal proportion of MK and FA precursors resulted in the AAM whose surface is shown in Figure 4c. This combination of precursors yields an AAM surface that is relatively homogeneous, with sporadic and randomly distributed pores through an amorphous (aluminosilicate gel) structure. The addition of wood ash to this system (Figure 4d) slightly disrupts its homogeneity. Furthermore, rod-shaped particles appear, growing up to a size of 1 micron. EDS results of the presented area show approximately the same Ca content for all three samples with added WA. Figure 4c indicates about a three times lower Ca content for AFA_50_MK_50_ compared to all other samples.

### 2.5. TEM Analysis

**Figure 5:**
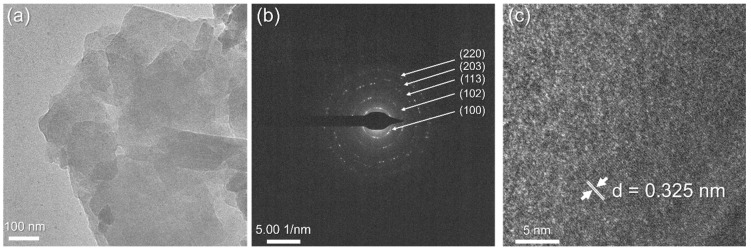
TEM analysis of AFA_50_MK_50_: (**a**) bright-field TEM micrograph, (**b**) SAED pattern, and (**c**) HR-TEM image.

The microstructure of the samples was analyzed using conventional transmission electron microscopy (TEM). Additionally, selected area electron diffraction (SAED) and high-angle annular dark-field imaging in scanning transmission electron microscopy (STEM) mode were conducted. Figure 5 presents the TEM analysis of the AFA_50_MK_50_ sample.

Figure 5a presents a bright-field TEM micrograph of the sample surface, where particles with well-defined edges are visible, along with a noticeable layered structure.

The crystal structure of the sample (Figure 5b) was confirmed using electron diffraction from a selected surface area. The presence of diffraction rings indicates that the material is polycrystalline. The measured diameters of the diffraction rings are 0.423 nm, 0.246 nm, 0.163 nm, 0.145 nm, and 0.122 nm, which correspond to (100), (102), (113), (203), and (220) crystallographic planes of quartz (PDF No. 00-033-1161), which is consistent with our XRD analysis. Figure 5c presents the corresponding HR-TEM image of AFA_50_MK_50_, showing good crystallinity with clearly defined planes. The interplanar distance of 0.325 nm matches well with the d-spacing for albite (PDF No. 01-078-1995).

**Figure 6:**
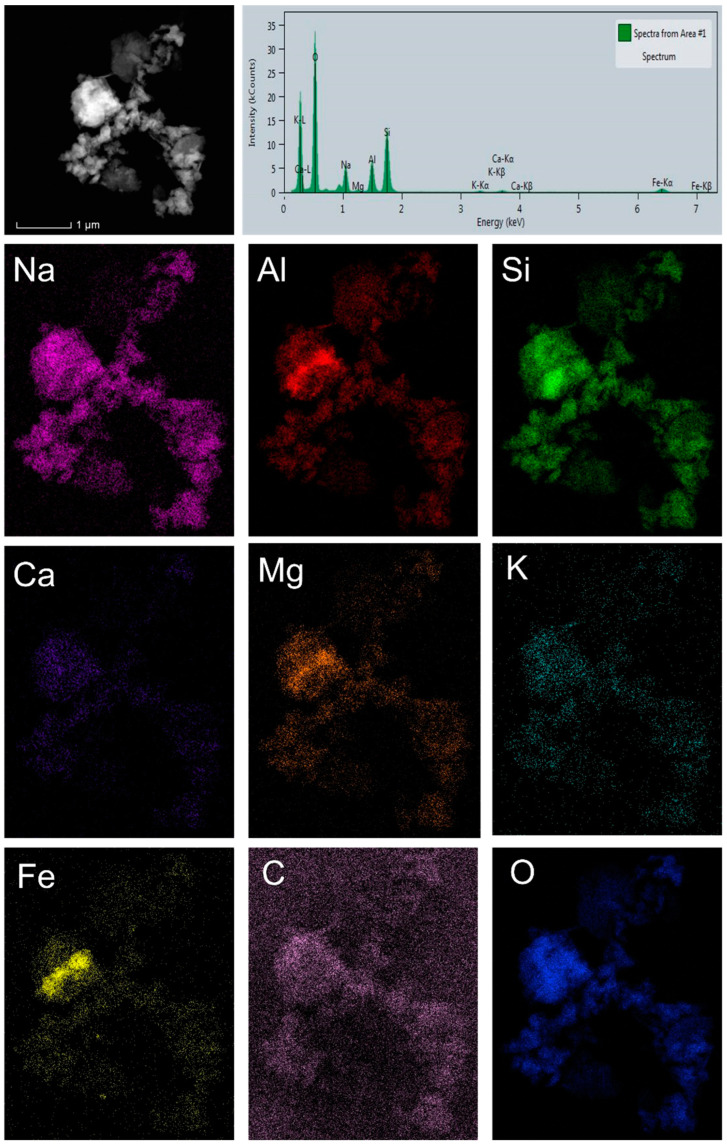
STEM/HAADF images with corresponding EDS mapping of AFA_50_MK_50_.

Figure 6 presents the STEM/HAADF images and the corresponding EDS mapping of AFA_50_MK_50_.

Figure 6 shows that the distribution of elements in the sample is generally uniform, with the exception of iron, which is concentrated in one part of the specimen.

**Figure 7:**
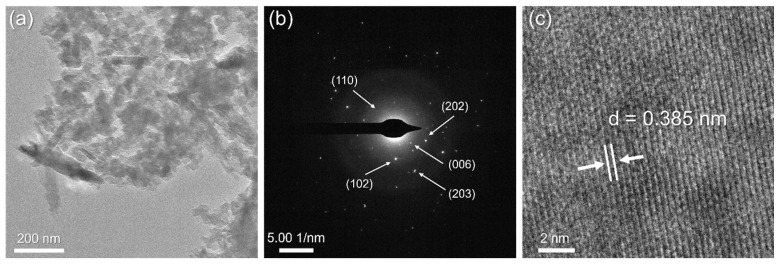
TEM analysis of AWA_10_FA_45_MK_45_: (**a**) bright-field TEM micrograph, (**b**) SAED pattern, and (**c**) HR-TEM image.

Figure 7 represents the TEM analysis of the AWA_10_FA_45_MK_45_ sample.

Based on Figure 7, the measured diameters of the diffraction rings are 0.324 nm, corresponding to the (006) crystallographic plane of muscovite (PDF No. 01-076-0928); 0.202 nm, corresponding to the (202) crystallographic plane of calcite (PDF No. 01-078-4616); and 0.244 nm, 0.232 nm, and 0.134 nm, corresponding to the (110), (102), and (203) crystallographic planes of quartz (PDF No. 00-033-1161), respectively. The HR-TEM image presented in Figure 7c indicates good crystallinity of the sample, with clearly defined planes. The measured interplanar spacing of 0.385 nm matches the characteristic d-values for the calcite crystal plane (PDF No. 01-078-4614).

**Figure 8:**
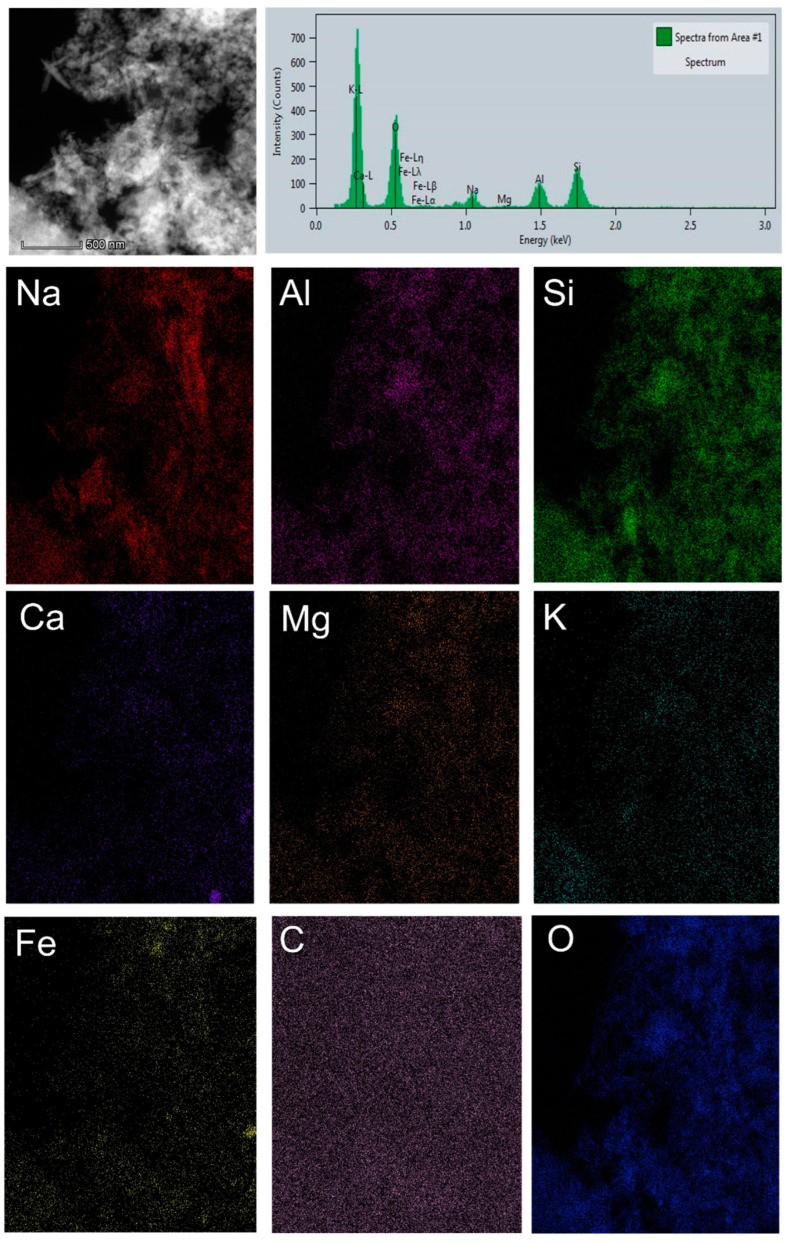
STEM/HAADF image with corresponding EDS mapping of AWA_10_FA_45_MK_45_.

Figure 8 shows the STEM/HAADF image of AWA_10_FA_45_MK_45_ with corresponding EDS mapping.

Based on the EDS maps (Figure 8), an even distribution of all elements on the surface of the sample can be observed.

### 2.6. Radiological Characterization of AAMs

Alkali-activated materials, made from industrial by-products, require careful consideration of their natural radioactivity levels. Evaluating the radioactivity of these materials is essential to determine the potential radiological risks they may pose to human health in both indoor and outdoor environments [14,33]. Understanding the radiation doses a person might be exposed to under specific conditions is crucial for evaluating the associated risks and potential effects on the human body. The activity concentration of naturally occurring radionuclides (^226^Ra, ^232^Th, and ^40^K) in the raw mixture and alkali-activated materials is presented in Table 2 and Table 3, respectively. Radium equivalent activity (*Ra*_eq_), external hazard index (*H*_ex_), external absorbed dose rate (*Ḋ*), and annual effective dose rate (*EDR*) were calculated to estimate potential health effects due to public exposure to natural radionuclides present in the raw mixture and their corresponding alkali-activated materials, as shown in Table 2 and Table 3.

It can be observed that the activity concentrations of ^226^Ra, ^232^Th, and ^40^K have lower values after the alkali activation process for all samples besides the activity concentration of ^226^Ra and ^40^K WA_10_FA_90_, and the calculated values of the gamma index of measured specific activities are lower in the alkali-activated samples. *Ra*_eq_ for all the tested samples is lower than 370 Bq/kg, which verifies that the evaluated hazard (gamma dose) connected with raw mixtures and AAMs containing ^226^Ra, ^232^Th, and ^40^K is under the limit of 1 mSv/y [34]. An additional essential radiological norm to estimate the suitability of materials is the external hazard index (*H*_ex_), which must be less than 1. All analyzed samples meet this condition, as their *H*_ex_ values are below the defined limit. However, the external absorbed dose rate (*Ḋ*) for the investigated raw mixtures exceed the average value of terrestrial outdoor gamma radiation for the human population. The average value of terrestrial outdoor gamma radiation is 60 nGy/h, determined by UNSCEAR (2000) [35]. In Findanchevski et al.’s research (2021) [36], in a different kind of FA, the external absorbed dose rate was in the range from 89.22 to 166.11 nGy/h. According to an investigation by Bošković et al. (2018), in alkali-activated red mud and metakaolin, the value of *H*_ex_ and *Ḋ* were 0.578 Bq/kg and 177.6 nGy/h, respectively [37]. Additionally, Sas et al. (2019) inspected alkali-activated fly ash, slag, and red mud, and radiological analysis of the different mixtures showed the values to be 41–57 Bq/kg for 226Ra, 34–57 Bq/kg for 232Th, and 336–490 Bq/kg for ^40^K [38].

The *Ḋ* values for the corresponding AAMs are within the range of 74.7 nGy/h for AFA_50_MK_50_ and up to 107.3 nGy/h for AWA_10_MK_90_. The values of the annual effective dose rate for all AAMs are below effective dose rate level of 1 mSv/y [39]. Lower values of *Ra*_eq_, *H*_ex_, *Ḋ*, and *EDR* for AAMs compared to those of the corresponding raw mixtures from which they were obtained are to be expected, as all these values clearly depend on the activity concentration of ^226^Ra, ^232^Th, and ^40^K. This was also demonstrated in the research by Fidanchevski et al. (2021) [36]. The activity concentration of naturally occurring radionuclides is consistent with the grain size [36] of the material, and since the ashes and metakaolin have smaller grain sizes [22], they are expected to have a higher content of radionuclides and therefore higher values of *Ra*_eq_, *H*_ex_, *Ḋ*, and *EDR*. This is one of the possible explanations for the obtained results of the mentioned parameters.

## 3. Conclusions

This study investigated the properties of alkali-activated materials (AAMs) made from Al- and Si-rich industrial by-products, such as fly ash (FA), which serves as a suitable alternative to metakaolin (MK) as a precursor of AAMs. Additionally, wood ash (WA) was included as a part of solid precursor in specific proportions due to its high calcium oxide content, which has been shown to significantly reduce the setting time of geopolymers. By employing advanced techniques like XRF, DRIFT, XRD, FESEM, TEM, and radiological assessments, the study provided crucial insights into their chemical composition, structural characteristics, and potential radiological risks. XRF and DRIFT analyses confirmed the consistent oxide content and the presence of functional groups, respectively. The DRIFT technique indicated successful geopolymer formation. XRD revealed a predominantly amorphous geopolymer gel matrix, with some crystalline phases identified in all samples. Most of them, such as quartz, mullite, and kaolinite, originated from the solid precursors and are not soluble under the applied conditions and activator. FESEM and TEM analyses demonstrated variations in microstructure, particle morphology, and crystallinity depending on the precursors used, further corroborating the XRD results. The proportion of fly ash (FA) in the mixture of metakaolin (MK), wood ash (WA), and FA significantly influences the physicochemical properties of alkali-activated materials (AAMs). An increase in the FA content typically enhances certain properties due to its high silica and alumina content, which plays a crucial role in geopolymerization. A higher FA content contributes to the formation of a dense amorphous geopolymer matrix and generally leads to a reduced porosity and a more homogeneous microstructure. However, the incorporation of WA might introduce heterogeneities due to high-Ca phases.

This study emphasized the importance of radiological evaluations to ensure the safe application of AAMs. All analyzed samples met safety conditions, as their *H*_ex_ values were below the defined limit. The absorbed dose rate (*Ḋ*) values for the corresponding AAMs ranged from 74.7 nGy/h for AFA_50_MK_50_ up to 107.3 nGy/h for AWA_10_MK_90_. The annual effective dose rate values for all AAMs were below the acceptable threshold of 1 mSv/y. These findings highlight the potential of AAMs as sustainable construction materials, provided their radiological impacts are carefully managed. Research efforts toward further optimizing the composition and production conditions of AAMs based on waste materials rich in Al and Si in this field are ongoing.

## 4. Materials and Methods

### 4.1. Preparation of Samples

For the synthesis of the alkali-activated materials, as a precursor, blends or mixtures of fly ash (FA), wood ash (WA), and metakaolin (MK) were used. Fly ash was sourced from the “Nikola Tesla” power plant in Obrenovac, Serbia; wood ash was derived from the combustion of wood in a domestic fireplace; and metakaolin was produced by thermally treating kaolin (local clay) at 750 °C in an air-atmosphere furnace, with a heating rate of 10 °C/min and a one-hour holding time at the target temperature. Alkali-activated materials were synthesized into a mixture of fly ash, wood ash, and metakaolin and an alkali activator solution consisting of sodium hydroxide (Sigma-Aldrich, St. Louis, MO, USA) and a sodium silicate solution (Interhem Company, Belgrade, Serbia). The mix design of solid precursors for the alkali activation process is presented in Table 4.

The concentration of the sodium hydroxide solution was 6 mol/dm^3^ (6M). The ratio of the liquid and solid phase was in the interval of 0.8–1.0 as a function of a structure in a different mixture. The precursors and alkali activator solution were combined, poured into molds, covered, and left to cure at room temperature for one day. Subsequently, the mixture was kept at 60 °C for two days, followed by approximately four weeks of curing at room temperature under controlled conditions. The alkali-activated materials were labeled in the following way: AMx,y, where M represents FA, WA, or MK and the subscript x, y indicates the percentage of the corresponding precursor in the solid raw mixture.

### 4.2. Method of Characterizations

#### 4.2.1. XRF Analysis

Chemical composition was conducted through X-ray fluorescence spectroscopy–XRF. The XRF analysis was performed with a wavelength dispersion (WD XRF) spectroscope ARL Perform X, manufactured by Thermo Scientific (Houston, TX, USA), with a power of 2500 W, 5 GN Rh X-ray tube, 4 crystals (AX03, PET, LiF200 and LiF220), two detectors (proportional and scintillation), and computer program UniQuant 5. The samples were quartered, dried at 105 °C, and calcined at 950 °C. For measurement purposes, a fused pellet was prepared, where 0.7640 g of the sample and 7.64 g of the flux (50% lithium tetraborate versus 50% lithium metaborate) were melted at 1100 °C. The estimated uncertainty of the measurement was 3.0–5.0%.

#### 4.2.2. DRIFT Analysis

The functional groups of all samples were determined through diffuse reflectance infrared Fourier transform (DRIFT) spectroscopy. The DRIFT spectra were obtained using the Perkin–Elmer FTIR spectrometer Spectrum Two (resolution—4 cm^−1^, mid-IR region from 4000 to 400 cm^−1^).

#### 4.2.3. XRD Analysis

Mineralogical characterization of AAMs was examined through X-ray diffraction (XRD) (Ultima IV Rigaku diffractometer (Rigaku, Tokyo, Japan)—Cu Kα1,2 radiation; generator voltage—40.0 kV; generator current—40.0 mA; range 2θ—5–80°; scanning step size—0.02°; scan rate—5°/min) [40,41].

#### 4.2.4. FESEM-EDS Analysis

Field emission scanning electron microscopy with energy-dispersive X-ray spectroscopy (FESEM-EDS, FEI Scios2, Dual Beam system, Thermo Fisher Scientific, Houston, TX, USA) was used for both the morphological and elemental characterization of the samples. Prior to imaging, double-sided copper tape was used to attach the powder to a sample holder. The samples were then sputter-coated with gold to make them conductive. The micrographs were taken at an acceleration voltage of 10 kV and a chamber pressure of around 9 × 10^−5^ Pa.

#### 4.2.5. TEM Analysis

The structural characterization of the AAM_S_ was achieved through transmission electron microscopy (TEM) in conventional and high-resolution modes, using an FEI Talos F200X microscope (Thermo Fisher Scientific, Waltham, MA, USA) operated at 200 keV. Electron diffraction on a selected surface was also performed. The samples for TEM investigation were prepared by dispersing the powder in ethanol, and after dispersion, a drop of the solution was placed on a carbon-coated copper grid and dried in air. Characteristic interplanar distances were determined from representative HR-TEM micrographs, using the ImageJ photo processing program [42].

#### 4.2.6. Radiological Characterizations

The activity concentrations of naturally occurring radionuclides (uranium and thorium series, 40K and 235U) in the AAM samples were measured using a high-purity germanium (HPGe) semiconductor detector. Details of the procedure are provided in our previous study [9]. Standards for energy and efficiency calibration of the spectrometer were determined using a certified solution of mixed gamma-emitting radionuclides (^241^Am, ^109^Cd, ^139^Ce, ^57^Co, ^60^Co, ^137^Cs, ^113^Sn, ^85^Sr, ^51^Cr, ^210^Pb, and ^88^Y), obtained from the Czech Metrology Institute (CMI) [43], in accordance with IAEA recommendations [44].

After the samples reached radioactive equilibrium, they were analyzed with spectra, recorded, and processed using Canberra’s Genie 2000 software. Corrections for background radiation, dead time, and coincidence summing effects were applied. All calculations were performed using Mathematica 5.2 software (Wolfram Research, Inc., Champaign, IL, USA).

To evaluate the potential health impacts from exposure to natural radionuclides in the investigated materials, parameters such as radium equivalent activity *Ra*_eq_ (Bq/kg), external hazard index *H*_ex_ (Bq/kg), absorbed gamma dose rate *Ḋ* (nGyh/h), and annual effective dose rate *EDR* (mSv/y) were calculated using the equations provided in Table 5.

Radium equivalent activity (*Ra*_eq_) is the index determined to obtain the amount of activities for the comparison of specific radioactivity of the samples containing various radionuclides, ^226^Ra, ^232^Th, and ^40^K. We analyzed the characteristics of the samples in order to estimate the potential of their use as building materials; for limiting the radiation dose from the building materials, we used the external hazard index (*H*_ex_). The value of this index must be less than 1.0, to remain under the radiation hazard limits, i.e., to retain the radium equivalent activity and annual dose below the allowed limits of 370 Bq/kg and 1 mSv, respectively [19].

The external gamma dose rate ($\overset{\cdot }{D}$, nGy/h) in air, measured at a height of 1 m above the ground, was determined based on the radionuclides ^226^Ra, ^232^Th, and ^40^K present in the analyzed samples. To evaluate potential health risks, the annual effective dose rate was calculated. This calculation used a conversion factor of 0.7 Sv/Gy to convert the absorbed dose in air to an effective dose, an indoor occupancy factor of 0.8 (reflecting that people spend about 80% of their time indoors), and an annual exposure duration of 8760 h (1 year), as recommended by UNSCEAR (1993) [46].

## Figures and Tables

**Table 1:** Chemical composition of alkali-activated materials.

Chem. Comp. (wt.%)	Na_2_O	MgO	Al_2_O_3_	SiO_2_	P_2_O_5_	SO_3_	K_2_O	CaO	TiO_2_	MnO	Fe_2_O_3_	As_2_O_3_	BaO	LOI * 950 °C
AWA_10_FA_90_	8.77	1.35	16.03	43.04	0.153	0.135	1.57	4.68	0.39	0.185	3.29	0.106	0.065	20.10
AWA_10_MK_90_	7.19	0.633	20.92	48.16	0.163	0.027	2.26	3.33	0.422	0.168	1.23	0.106	0.047	15.24
AFA_50_MK_50_	6.93	0.92	20.37	53.37	0.03	0.02	1.20	1.49	0.44	0.03	2.59	0.16	0.04	12.29
AWA_10_FA_45_MK_45_	8.61	1.02	18.03	45.06	0.14	0.08	1.78	3.75	0.40	0.16	2.29	0.01	0.06	18.39

* loss on ignition.

**Table 2:** Activity concentration of naturally occurring radionuclides ^226^Ra, ^232^Th, and ^40^K and *Ra*_eq_, *H*_ex_, *Ḋ*, and *EDR* for raw mixtures.

Raw Mixture	^226^Ra	^232^Th (^228^Ac)	^40^K	*I*_γ_	*Ra*_eq_, Bq/kg	*H*_ex_, Bq/kg	*Ḋ*, nGy/h	*EDR*, mSv/y
WA_10_FA_90_	83.1 ± 6.6	58.8 ± 3.6	465 ± 24	0.726	203.0	0.548	93.3	0.458
WA_10_MK_90_	130.7 ± 10.7	76.4 ± 4.6	660 ± 34	1.038	290.8	0.785	134.1	0.658
FA_50_MK_50_	128.3 ± 10.6	81. 4 ± 4.8	393 ± 21	0.966	275.0	0.743	124.8	0.612
WA_10_FA_45_MK_45_	125.9 ± 9.3	73.5 ± 4.7	635 ± 33	0.999	279.9	0.756	129.0	0.633

**Table 3:** Activity concentration of naturally occurring radionuclides ^226^Ra, ^232^Th, and ^40^K and *Ra*_eq_, *H*_ex_, *Ḋ*, and *EDR* for AAMs.

AAMs	^226^Ra	^232^Th (^228^Ac)	^40^K	*I*γ	*Ra*_eq_, Bq/kg	*H*_ex_, Bq/kg	*Ḋ*, nGy/h	*EDR*, mSv/y
AWA_10_FA_90_	95.9 ± 8.7	55.7 ± 3.9	486 ± 26	0.760	213.0	0.575	98.2	0.482
AWA_10_MK_90_	85.7 ± 6.4	68.8 ± 4.6	627 ± 33	0.839	232.4	0.628	107.3	0.526
AFA_50_MK_50_	66.0 ± 5.5	54.5 ± 3.4	271 ± 15	0.583	164.8	0.445	74.7	0.367
AWA_10_FA_45_MK_45_	72.3 ± 6.3	47.0 ± 3.2	397 ± 21	0.608	170.1	0.459	78.3	0.384

**Table 4:** Mix design of solid precursors.

Raw Mixture	Precursors		
	WA (%)	FA (%)	MK (%)
WA_10_FA_90_	10	90	0
WA_10_MK_90_	10	0	90
FA_50_MK_50_	0	50	50
WA_10_FA_45_MK_45_	10	45	45

**Table 5:** Equations for dose calculation for investigated materials.

Dose Calculation	Formula	References
Gamma index	$I_{\gamma }=\frac{A_{Ra}}{300Bq/kg}+\frac{A_{Th}}{200Bq/kg}+\frac{A_{K}}{3000Bq/kg}\;\leq 1$	[33]
Radium equivalent activity	${Ra}_{eq}=A_{Ra}+1.43A_{Th}+0.077A_{K}$	[33]
External hazard index	$H_{ex}=\frac{A_{Ra}}{370}+\frac{A_{Th}}{259}+\frac{A_{K}}{4810}\leq 1$	[33]
External absorbed gamma dose rate	$\overset{\cdot }{D}=0.462A_{Ra}+0.604A_{Th}+0.0417A_{K}$	[33]
Annual effective dose rates	*EDR*(mSv) = $\overset{\cdot }{D}$ (nGy/h) × 8760·(h/y) × 0.8 × 0.7 (Sv/Gy) 10^−6^	[33,45]

A_Ra_, A_Th_, and A_K_ are activities in Bq/kg of ^226^Ra, ^232^Th, and ^40^K, respectively.

## Data Availability

The data presented in this study are available on request from the corresponding author.
